# The benefits of contrast-enhanced ultrasound and why we don’t use more contrast-enhanced ultrasound in clinical practice in the United Kingdom

**DOI:** 10.1093/bjr/tqag025

**Published:** 2026-02-03

**Authors:** Gibran Timothy Yusuf, Paul Singh Sidhu

**Affiliations:** King’s College Hospital NHS Foundation Trust, London, United Kingdom; King’s College Hospital NHS Foundation Trust, London, United Kingdom

**Keywords:** ultrasound, contrast-enhanced ultrasound, training

Contrast-enhanced ultrasound (CEUS) should not be considered a new modality given its presence in imaging and radiology for over 30 years.^1^ There has been a significant refinement of the technique over the years, particularly during the early period, with a sophisticated technique emerging and being applied in daily clinical practice. Multiple practice guidelines have been developed in the international arena, pioneered and led by the European Society and Federation of Ultrasound in Medicine and Biology (EFSUMB).^2^ The most widely recognized role of CEUS is in characterizing focal liver lesions (FLL), with a 20-year review of the literature citing hepatic use as the most published indication.^3^^,^^4^ This complies with the recommendation of the National Institute of Clinical Excellence (NICE) in the United Kingdom (UK) for assessing indeterminate liver lesions and the American College of Radiologists (ACR) for use in assessing hepatocellular carcinoma (HCC) in chronic liver disease. Although CEUS evaluation of FLL constitutes the main imaging indication, a myriad of applications outside the liver exists, including renal and testicular lesion characterization as well as interventional use and intracavitary use, amongst others.^5^

Contrast-enhanced ultrasound as a modality provides a number of benefits for patient imaging. Inherent to ultrasound is the absence of radiation, imaging in real-time and portability, allowing it to be performed by the bedside, which is particularly important for patients in intensive care. A key benefit is the ability to perform the test and gain greater diagnostic information in a single sitting. This has been recognized in a qualitative study as one of the key benefits, particularly as there is the ability to provide scan results in 80%-90% of cases on the same day as well as the perceived decreased waiting time for a diagnostic result compared to CT (computed tomography)/MRI (magnetic resonance imaging).^6^ The same study notes that approximately 20-30 minutes are required for CEUS; whilst this is not directly compared to the time taken for another examination, it may be considerably shorter than for MRI, especially if a hepatocyte-specific contrast agent is used. Notably, for children, ultrasound avoids the burden of radiation, a possible risk factor for the development of cancer.^7^ Although ultrasound is subject to operator variability, in experienced hands using multiplanar dynamic imaging to obtain a diagnostic outcome is readily achievable; the biggest danger to ultrasound is the inexperienced operator outside their skill set. Ultrasound contrast agents are true blood pool agents, allowing real-time assessment of overlapping phases of imaging, unlike the cross-sectional techniques of MRI or CT. It has been shown that CEUS is as effective as CT or MRI for characterization of FLL, differentiating benign from malignant, and metastasis from HCC accurately.^8^ A CEUS examination has inherent superior resolution to CT and MR imaging, which has led to applications in renal cystic lesion grading, assessing testicular lesions and has considerable usefulness in interventional radiology.^5^ A number of centres across the United Kingdom have successfully implemented CEUS, but data on this remain scarce. A study from 2007 cites the sensitivity for diagnosing malignancy at 95% and states that CEUS is suitable for a district general hospital where imaging resources may be limited and can be implemented with relatively small expense.^9^

Ultrasound contrast agents have had an enviable safety record, established over a long period of clinical practice, with the largest study of 463 464 doses indicating an adverse incidence rate of 0.034%^10^ lower than that of iodinated contrast, and have been shown to be safe in children.^11^ With costs an important criterion for all healthcare systems, CEUS has demonstrated cost effectiveness, particularly for incidental FLL, with a cost analysis proposing up to £2.2 million in savings per year if FLL were imaged with CEUS as the first-line investigation following the baseline ultrasound.^12^ The importance of CEUS in establishing the benign FLL prevents escalating costs associated with recurrent attendances and diagnostics in establishing the absence of malignancy.^13^

Contrast-enhanced ultrasound, however, also has limitations, as per any modality. As CEUS is an adjunct to conventional ultrasound, it suffers from similar limitations. Most importantly, an appropriate acoustic window is needed, meaning overlying gas, and lack of depth penetration (including in obesity or marked hepatic steatosis) render CEUS ineffective. It is also important to note there is a learning curve with CEUS, and there is the risk of artificial microbubble attrition from continuous scanning leading to potential misdiagnosis, especially in FLL. Also, although considered a safe modality, the potential for an anaphylaxis reaction exists and requires appropriate equipment, rendering small, remote areas a potential risk to undertake CEUS if lone scanning.

Disappointingly the application of CEUS within the United Kingdom has remained relatively scant despite high prevalent use in Europe and China, with recent licensing increasing the use in the United States. This is demonstrated with a bibliographic review over 20 years showing China and Italy to dominate whilst the United Kingdom lies in seventh position.^3^ Whilst this may reflect academia, it provides an element of reflection on practice. Personal communication from industry shows that the use of SonoVue^TM^ (Bracco, Italy) in the United Kingdom is restricted to only 60 radiology accounts in the United Kingdom compared to over 200 for use in cardiology departments. Whilst in comparison CEUS is estimated to be used in 10%-15% of ultrasound examinations in China. Part of the reason for the discrepancy seen within Europe is that ultrasound is commonly performed by internal medicine physicians and speciality physicians rather than radiologists or sonographers, allowing for greater autonomy, unlike the United Kingdom, where examinations are prescribed via a referrer to a radiology department. Several perceived barriers exist and can be divided into structural, clinical or cultural, none of which are insurmountable.

Structural barriers occur at a systemic level, either on a local or national basis. Despite a wide variety of CEUS applications, there are limited licensing indications (breast, liver, cardiac and vascular uses), and NICE approval only exists for FLL. To alter licensed indications is an enormous and costly undertaking, and there would need to be sufficient financial return for the industry to undertake this, thereby limiting the ability of NICE to approve further uses. This is despite the wide variety of off-label uses, including one of the commonest utilities, in renal cysts. The narrow range of indications can make the proposal to start a CEUS service difficult, as local regulatory boards are often reluctant to provide ongoing approval for off-label use, such as renal cyst assessment—a dominant use for CEUS. This is compounded by cost pressures within the National Health Service (NHS). Although CEUS provides evidence of cost effectivity compared to CT or MR imaging, the initial outlay^12^ and recurring expense for the contrast agent and enabling software for existing ultrasound equipment are sometimes considered unacceptable, and the investment itself may require business cases for approval. Sonographers are restricted in the application of CEUS, as the ultrasound contrast agent is considered a pharmaceutical drug and requires prescription–often requiring either a patient group directive or direct prescribing from a doctor. This is particularly challenging again given the large volume of off-label utilities; sonographers will be limited to using CEUS for licensed applications only.

Ultrasound is a clinical technique and requires manual skill in addition to diagnostic knowledge. Ultrasound is well practised by sonographers, but the emphasis in training for radiologists is in cross-sectional imaging and other modalities, which can lead to a decline in sonographic skills, including image optimization. Unlike other modalities, in CEUS, the image acquisition and quality of examination are heavily operator dependent and directly influence the report and clinical value of the study. The decline of radiologist training in conventional ultrasound raises concern over the ability to perform advanced techniques reliably. Whilst training recommendations exist for the practice of CEUS,^14^ regulatory approval, time constraints and funding often prevent uptake. The majority of CEUS practice is restricted to university teaching hospitals, limiting availability for patients and compounding the limitations of training access and regular practice to improve skill sets. Lack of funding and staffing results in difficulty in obtaining permission to attend courses to upskill and develop knowledge. In addition, with limited regulatory approval and early practice mainly focusing on problem solving in FLL, numbers of patients are limited in individual departments, thereby limiting training opportunities.

Cultural issues perhaps predominate for low uptake of CEUS within the United Kingdom. With the exponential growth in cross-sectional imaging outstripping the growth in radiologist numbers,^15^ ultrasound risks becoming sidelined by radiologists in favour of CT and MRI. The majority of ultrasound in the United Kingdom is now provided by sonographers, despite increasing interest from clinical specialities such as hepatology, intensive care and emergency medicine. As a result, there is often reluctance of radiologists to undertake ultrasound, with the potential perception of ultrasound as time consuming and impractical compared to CT or MRI, which provides relative uniformity in imaging for interpretation and reported remotely. This perception is often propagated to resident doctors who carry forth the same belief. This is compounded by the fear that can be associated with learning any new technique with the risk of suboptimal imaging, further precipitating the desire for cross-sectional imaging. It is also important to be aware that many clinical teams are not aware of CEUS and by default request CT/MR imaging, feeling uncertain about the validity of results from ultrasound or CEUS. This is compounded by the lack of experience and confidence of the radiologist who does not understand the benefits of CEUS. A robust CEUS service requires more than a single operator to allow for contingency; however, with difficulty in training, it may require an individual to start, promote and run the service, requiring extensive commitment and prior paperwork, generating further hesitancy.

Whilst the problems the United Kingdom faces in the adoption of CEUS are considerable, they are not insurmountable. Ultimately the benefits of CEUS present the most compelling evidence, with cost- effectiveness a key part forming the basis of any business case to be presented. In the current financial climate, presentation of the cost reduction is a strong argument,^12^ this exists not just for directly lower costs compared to CT/MR imaging^16^ but also in reducing the number of steps in the patient journey and timeline before definitive management or discharge. The prevention of further cross-sectional imaging by CEUS also helps to improve growing waiting lists.^15^ Even if the utility of CEUS is restricted to licensed applications, the significant cost reduction remains apparent. When coupled with the safety of CEUS and the ability for the ultrasound contrast agent to be used in departments other than radiology (e.g. cardiology), the case for the introduction of a CEUS is irrefutably strong. When considering clinical skills and education, there is a growing recognition of CEUS and development of courses, with increasing prevalence in general radiology meetings on national and international levels. In addition, virtual congresses and webinars further the availability and reach of educational material; the use of social media has been shown to be key to the dissemination of education. The digital era also enables easy access worldwide to senior clinicians in the field for potential mentoring; the result is widespread availability of education allowing upskilling. The rise of CEUS may still be met with reluctance due to time pressures, but it is expected that sonographers and clinicians from other specialities will increasingly uptake the technique with a snowball effect. The technique may be more commonplace outside radiology. It is essential radiologists also recognize the value of CEUS and appreciate the greater benefits of the techniques and have an opportunity to lead in this field.^17^ Individuals aiming to become involved in the service will have to take a number of practical steps after financial and governance approvals. These will include a robust service likely involving more than a single individual, for which the inclusion of a radiologist is helpful not only for prescribing the contrast agent but also for medical knowledge and that of comparative imaging. It is also valuable to engage ultrasound applications specialists to ensure the optimal equipment settings. Continued education is essential, along with attendance at courses, and literature evaluation is needed. The authors suggest that in the digital age it is also easier to seek the advice of experts more readily than previously and is highly recommended.

In conclusion, with increasing pressure on cross-sectional modalities and increasing cost pressures, CEUS offers an alternative which can be performed safely and accurately. Regulatory approvals limiting growth of the technique represent a source of irony given CT and MR contrast agents are licensed for whole-body use, but ultrasound contrast agents are limited to specific organs despite systemic circulation.^18^ The increasing use of CEUS across the world is clearly apparent; however, the United Kingdom risks falling behind due to a number of barriers. With strong motivation, appropriate governance, adequate training and robust departmental infrastructure, CEUS in the United Kingdom has the potential to overcome these barriers and excel.
